# Disease Outcomes After Segmental Resection of Colonic Crohn’s Disease: A Retrospective Multicenter Study

**DOI:** 10.1093/ibd/izaf252

**Published:** 2025-12-22

**Authors:** George Salem, Christopher A Bouvette, Cristian Hernandez-Rocha, Oscar Hernandez Dominguez, James Conner, Mark Silverberg, Benjamin L Cohen, Stefan Holubar, Mark Lazarev

**Affiliations:** Section of Digestive Diseases and Nutrition, Department of Medicine, University of Oklahoma College of Medicine, Oklahoma City, OK 73104, United States; Division of Gastroenterology, Department of Medicine, Johns Hopkins University School of Medicine, Baltimore, MD 21287, United States; Section of Digestive Diseases and Nutrition, Department of Medicine, University of Oklahoma College of Medicine, Oklahoma City, OK 73104, United States; Zane Cohen Centre for Digestive Diseases, Lunenfeld Tanenbaum Research Institute, Mount Sinai Hospital, Toronto, ON M5T 3L9, Canada; Division of Gastroenterology and Hepatology, University of Toronto Temerty Faculty of Medicine, Toronto, ON M5T 3L9, Canada; Department of Gastroenterology, Faculty of Medicine, Pontificia Universidad Católica of Chile, 8380453 Santiago, Chile; Zane Cohen Centre for Digestive Diseases, Lunenfeld Tanenbaum Research Institute, Mount Sinai Hospital, Toronto, ON M5T 3L9, Canada; Zane Cohen Centre for Digestive Diseases, Lunenfeld Tanenbaum Research Institute, Mount Sinai Hospital, Toronto, ON M5T 3L9, Canada; Digestive Diseases Institute, Cleveland Clinic Medical Center, Cleveland, OH 44195, United States; Department of Colorectal Surgery, Cleveland Clinic Foundation, Cleveland, OH 44195, United States; Division of Gastroenterology, Department of Medicine, Johns Hopkins University School of Medicine, Baltimore, MD 21287, United States

**Keywords:** Crohn’s disease, inflammatory bowel disease, segmental colectomy, postoperative IBD recurrence

## Abstract

**Background:**

Colonic surgery for Crohn’s disease (CD) frequently involves sparing uninvolved segments of the colon. Few studies have assessed recurrence rates after segmental colectomy (SC). The aim of this study was to determine the rate of and identify the risk factors for postoperative CD recurrence.

**Methods:**

This was a multicenter retrospective study from 3 tertiary inflammatory bowel disease (IBD) referral centers of CD patients who underwent SC between 2000 and 2019. We defined endoscopic recurrence as the presence of ulcers in the remaining colon upon postoperative colonoscopy.

**Results:**

A total of 108 patients were included. Sixty-nine (63.9%) patients had evidence of postoperative CD endoscopic recurrence. Age at surgery <40 years and disease duration ≤156 months predicted an increased likelihood for postoperative recurrence (odds ratio [OR], 2.43; *P =* .031 and OR, 3.29; *P =* .005, respectively), whereas abdominal perineal resection (OR, 0.21; *P =* .005), indication for SC of malignancy (OR, 0.14; *P =* .016), and postoperative use of tumor necrosis factor α (TNFα) inhibitor for prophylactic purposes (OR, 0.38; *P =* .040) negatively predicted disease recurrence. Disease duration ≤156 months (OR, 2.86; *P =* .039) and postoperative TNFα inhibitor prophylaxis remained significant (OR, 0.26; *P =* .013) upon multivariable modeling.

**Conclusion:**

Although high rates of recurrence persist within the postoperative phase of SC for CD, the postoperative use of TNFα inhibitor for prophylactic purposes for a subset of patients may promote a more durable endoscopic remission.

Key messages

- Intestine-sparing segmental colectomy is preferred for operative management of Crohn’s disease, although there are little data on postoperative course and rates of recurrence.
- The prophylactic use of anti-tumor necrosis factor α inhibitors is protective against disease recurrence following segmental colectomy.
- High rates of postoperative recurrence after segmental colectomy should prompt providers to consider a more aggressive approach to medical therapy across peri- and postoperative care.

## Introduction

Crohn’s disease (CD) bears significant morbidity in forms of hospitalization, healthcare spending, and decreased quality of life.^1–4^ Furthermore, operative intervention, repeat intervention(s), and long-term sequelae carry psychosocial hardship and lifelong health effects.^3^^,^^5^^,^^6^ A prior meta-analysis showed a near 50% risk of intestinal resection within 10 years of CD diagnosis.^7^ The advent of immunomodulators and biologics is thought to provide a potentially more durable biologic and clinical perioperative remission.^8^^,^^9^

The operative approach for colitis associated with Crohn’s has moved away from subtotal or total abdominal colectomy for Crohn’s patients with focal active disease or dysplasia. European Crohn’s and Colitis Organization and European Society of Colo-Proctology consensus guidelines advocate for segmental colectomy (SC) in approach to focal disease of less than one-third of the colon, or conditionally when 2 widely separated locations are affected.^10^ However, there are a lack of data describing the risk of recurrence after SC, or the benefit of proactive postoperative medical management. This is further complicated by demonstrations of earlier recurrence after SC in certain patients, without difference in need for permanent stoma.^11–13^ We aimed to assess rates of CD recurrence and predictors thereof in patients undergoing SC.

## Methods

This was a retrospective multicenter study of CD patients receiving care at 3 tertiary IBD referral centers between 2000 and 2019. Cases were recruited through review of center-specific pathology and surgery databases. We confirmed operative technique by operative and pathology reports. SC was defined as sigmoidectomy, left hemicolectomy, abdominal perineal resection (APR), transverse colectomy, or lower anterior resection. In certain instances, index resection did not fit within the previous cited conventional definitions of SC (ie, extended or multifocal resection) and thus were captured as “mixed approach.” We included patients with postoperative surveillance colonoscopy at 6 months or later.

Variables of interest included biologic characteristics, disease activity, surveillance colonoscopy findings with pathology reports, pre- and postoperative medical therapy, and subsequent treatment(s). We defined preoperative medical therapy by those patients receiving a tumor necrosis factor α (TNFα) inhibitor, immunomodulator, other biologics or 5-aminosalicylate in the 12 months prior to SC. Similarly, we defined immediate postoperative management across the 12 months following SC—this was holistic, including all patients with either a proactive prophylactic or reactive start of medical therapy. As a subset of this, postoperative anti-TNFα prophylaxis was defined as anti-TNFα inhibitors initiated or resumed within 12 months after SC in the absence of clinical or endoscopic recurrence. A similar subcohort was defined to include alternative biologic CD therapies (eg, vedolizumab, ustekinumab).

We defined postoperative endoscopic recurrence as presence of ulcers, stricture, or >50% circumferential erosion at the surgical anastomosis. Postoperative endoscopic scores were not available for review. Our primary endpoint was recurrence rate at time of first colonoscopy. The cohort was limited to patients with first colonoscopy at >6 months after operative intervention. Chi-square and 2-factor *t* test assessed cohort characteristics across categorical and continuous variables, respectively. The *F* test of equality of variances assessed distribution prior to choice of Student’s or Welch’s *t* test. Univariable logistic regression assessed those factors that predicted disease recurrence. Multivariable stepwise logistic regression analysis calculated adjusted odds ratios (ORs) through modeling of variables with at least a modest correlation (*P* < .1). A *P* value <.05 was considered statistically significant. Kaplan-Meier hazard modeling described time to recurrence across the cohort. Subanalysis with time-to-event modeling across the recurrence strata assessed time to repeat operative intervention. The data underlying this article cannot be shared publicly for the privacy of individuals that participated in the study.

## Results

The initial cohort was 160 potentially eligible patients with colonic and ileocolonic CD who had undergone SC. After reviewing clinical course, 52 patients were excluded due to first postoperative colonoscopy at <6 months. The remaining cohort demonstrated appropriate follow-up interval and was analyzed. A total of 108 patients were included (49 males [45.4%]), with a median age at diagnosis of 23 years (range 6-74 years). Disease location was limited to the colon in 65 (60.2%) patients. Fifty-six (51.9%) patients had stricturing disease behavior, and 33 (30.6%) patients had evidence of perianal disease at the time of surgery. The median disease duration at the time of colonic surgery was 156 months (range 0-540 months). Forty-four (40.7%) patients had historic operative intervention. The type of segmental resection included sigmoidectomy, with 33 (30.6%); left hemicolectomy, with 20 (18.5%); APR, with 18 (16.7%); transverse colectomy, with 9 (8.3%); lower anterior resection, with 7 (6.5%); and a mixed approach, with 21 (19.4%). The median time between colonic surgery and the first endoscopic evaluation was 17 months (range 7-252 months). The median follow-up time after surgery was 70 months (range 13-276 months) (Table 1).

**Table 1.** Cohort characteristics and chi-square and 2-factor *t* test results (n = 108).

Characteristic	Recurrence	No recurrence	Aggregate	*P* value
**Sample**	69 (63.9)	39 (36.1)		
**Sex**				
** Female**	40 (58.0)	19 (48.7)	59 (54.6)	.354
** Male**	29 (42.0)	20 (51.3)	49 (45.4)	
**Age at diagnosis, y**	21 (6-63)	26 (10-74)	23 (6-74)	.349
**Disease behavior**				
** B1**	9 (13.0)	5 (12.8)	14 (13.0)	.974
** B2**	35 (50.7)	21 (53.8)	56 (51.9)	.755
** B3**	25 (36.2)	13 (33.3)	38 (35.2)	.762
** p**	16 (23.2)	17 (43.6)	33 (30.6)	.027^a^
**Location**				
** L1**	0 (0)	1 (2.6)	1 (1.0)	.181
** L2**	48 (69.6)	17 (43.6)	65 (60.2)	.008^a^
** L3**	21 (30.4)	21 (53.8)	42 (38.9)	.017^a^
** L4**	2 (2.9)	0 (0)	2 (1.9)	.283
**Age at SC, y**	35 (14-66)	47 (18-74)	39 (14-74)	.003^a^
**Disease duration at surgery, mo**	108 (0-540)	180 (0-528)	156 (0-540)	.014^a^
**Operative history**	26 (37.7)	18 (46.2)	44 (40.7)	.389
**Indication for surgery**				
** Refractory stricture**	38 (55.1)	16 (41.0)	54 (50.0)	.161
** Fistula**	23 (33.3)	10 (25.6)	33 (30.6)	.405
** Refractory to medical therapy**	12 (17.4)	8 (20.5)	20 (18.5)	.688
** Malignancy**	2 (2.9)	7 (17.9)	9 (8.3)	.007^a^
** Perforation**	8 (11.6)	3 (7.7)	11 (10.2)	.520
**Operative approach**				
** Left hemicolectomy**	14 (20.3)	6 (15.4)	20 (18.5)	.528
** Sigmoidectomy**	22 (31.9)	11 (28.2)	33 (30.6)	.690
** APR**	6 (8.7)	12 (30.8)	18 (16.7)	.003^a^
** LAR**	3 (4.3)	4 (10.3)	7 (6.5)	.231
** Transverse colectomy**	6 (8.7)	3 (7.7)	9 (8.3)	.856
** Mixed**	16 (23.2)	5 (12.8)	21 (19.4)	.191
** Time to colonoscopy, mo**	17 (7-152)	14 (7-252)	17 (7-252)	.683
**Preoperative management (12 mo)**				
** Anti-TNFα**	29 (42.0)	17 (43.6)	46 (42.6)	.875
** Immunomodulators**	22 (31.9)	12 (30.8)	34 (31.5)	.905
** Other biologics**	9 (13.0)	1 (2.6)	10 (9.3)	.071
** 5-aminosalicylate**	13 (18.8)	6 (15.4)	19 (17.6)	.650
**Postoperative management (12 mo)^b^**				
** Anti-TNFα**	24 (34.8)	15 (38.5)	39 (36.1)	.702
** Immunomodulators**	18 (26.1)	9 (23.1)	27 (25.0)	.729
** Other biologics**	11 (15.9)	3 (8.7)	14 (13.0)	.220
** 5-aminosalicylate**	12 (17.4)	5 (12.8)	17 (15.7)	.531
** Postoperative TNF prophylaxis^c^**	11 (15.9)	13 (33.3)	24 (22.2)	.037^a^
** Postoperative biologics prophylaxis^c^**	3 (4.3)	1 (2.6)	4 (4.0)	.637
** Repeat surgery**	20 (29.0)	3 (7.7)	23 (21.3)	.009^a^
** Duration to repeat surgery, mo**	53 (11-162)	89 (57-121)	56 (11-162)	.540

Values are n (%) or median (range).

Abbreviations: APR, abdominal perineal resection; CD, Crohn’s disease; LAR, lower anterior resection; SC, segmental colectomy; TNF, tumor necrosis factor

a A *P* value <.05 was considered statistically significant.

b Postoperative management: CD-directed medical therapy utilized in the 12 months after surgery (regardless of indication, proactive or reactive use).

c Postoperative TNF/biologics prophylaxis: TNF/other biologic class CD medications initiated or resumed within 12 months of surgery in the absence of clinical or endoscopic recurrence.

**Figure 1.**
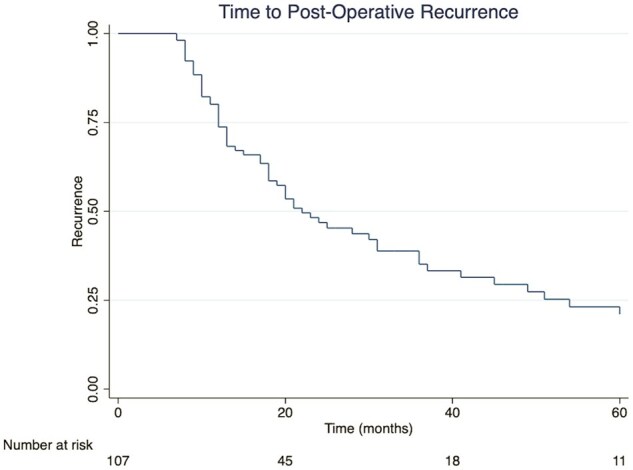
Time-to-event modeling of postoperative Crohn’s disease recurrence.

**Figure 2.**
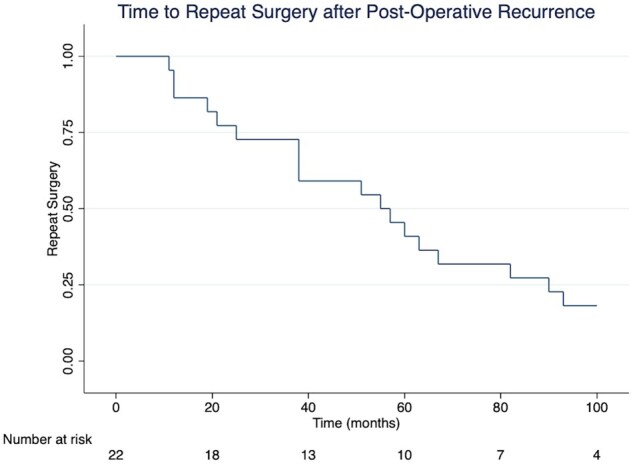
Within those patients with postoperative recurrence, time-to-event modeling of progression to repeat Crohn’s disease–directed surgery.

Sixty-nine (63.9%) patients had evidence of endoscopic recurrence at time of first colonoscopy. Median time to postoperative recurrence was 17 months (range 7-152 months) (Figure 1). Patients with endoscopic recurrence were younger at the time of colonic resection (median age 35 years vs 47 years) compared with patients who did not have evidence of recurrence (*P =*.003). The original indications for surgery were refractory stricture in 55.1% vs 41.0% (*P =* .161) and malignancy in 2.9% vs 17.9% (*P =* .007), comparing disease recurrence and no recurrence, respectively. Rates of preoperative TNFα inhibitor use were no different between groups (42% vs 43.6%; *P =* .875). Furthermore, rates of postoperative TNFα inhibitor use (34.8% vs 38.5%; *P =* .702) or other biologic use (15.9 vs 8.7%; *P =* .220) were no different between groups. However, the proportion of patients who received postoperative TNFα inhibitor prophylaxis was greatest in patients who did not have postoperative recurrence (33.3% vs 15.9%; *P =* .037). The overall surgical recurrence rate was 21.3%, of which 87% was attributed to CD recurrence. Those patients with postoperative CD recurrence were more likely to require repeat operative intervention (29.0% vs 7.7%; *P =* .009) (Table 1). Hazard modeling demonstrates a median time to repeat operative intervention of 53 months (range 11-162 months) across those patients with postoperative CD recurrence (Figure 2). Among the 3 (7.7%) patients without documented endoscopic recurrence who underwent repeat surgery, the indications were noninflammatory in nature.

Univariable regression demonstrated age at surgery <40 years (OR, 2.43; *P =* .031) and disease duration ≤156 months (OR, 3.29; *P =* .005) to predict increased likelihood for postoperative recurrence. APR (OR, 0.21; *P =* .005), indication for SC of malignancy (OR, 0.14; *P =* .016), and use of postoperative TNFα inhibitor prophylaxis (OR, 0.38; *P =* .040) negatively predicted disease recurrence (Table 2). On multivariable regression, disease duration ≤156 months (OR, 2.86; *P =* .039) remained significant. Furthermore, postoperative TNFα inhibitor prophylaxis remained significant (OR, 0.26; *P =* .013), independently predicting a lower risk of postoperative CD recurrence (Table 2).

**Table 2.** Predictors of postoperative recurrence, and univariable and multivariable logistic regression.

Characteristic	Univariable modeling		Multivariable modeling	
	OR (95% CI)	*P* value	OR (95% CI)	*P* value
**Perianal disease**	0.41 (0.18-0.94)	.036^a^	0.51 (0.18-1.44)	.205
**Age at surgery <40 y**	2.43 (1.08-5.45)	.031^a^	1.81 (0.70-4.67)	.221
**Disease duration ≤156 mo**	3.29 (1.43-7.58)	.005^a^	2.86 (1.06-7.75)	.039^a^
**APR**	0.21 (0.07-0.63)	.005^a^	0.35 (0.08-1.45)	.149
**SC for malignancy**	0.14 (0.03-0.69)	.016^a^	0.26 (0.04-1.74)	.165
**Postoperative TNF prophylaxis^b^**	0.38 (0.15-0.96)	.040^a^	0.26 (0.09-0.76)	.013^a^

All variables assessed under univariable modeling, only those with at least a modest correlation (*P* < .1) were reported and included in multivariable modeling.

Abbreviations: APR, abdominal perineal resection; CI, confidence interval; CD, Crohn’s disease; OR, odds ratio; TNF, tumor necrosis factor.

a A *P* value <.05 was considered statistically significant.

b Postoperative TNF prophylaxis: TNF class CD medications initiated or resumed within 12 months of surgery in the absence of clinical or endoscopic recurrence.

## Discussion

The major finding of the current study is the high rate of endoscopic recurrence in the postoperative phase of SC for CD, and this in turn increases the need for repeat resection surgery. The postoperative use of TNFα inhibitor for prophylactic purposes independently predicted lower rates of postoperative CD recurrence. Collectively, this evidence supports the overall concept that while SC may provide improved quality of life as compared with total colectomy, the risk for recurrence is high and needs to be medically managed.

We demonstrate an overall postoperative recurrence rate of 64% at a median of 17 months after surgery. This is in contrast to the historically lower rates of recurrence demonstrated for patients undergoing total colectomy or proctocolectomy with permanent ileostomy.^12–14^ Further, this is strikingly higher than rates of postoperative recurrence after CD-directed ileocecal resection.^15^ We suspect that those patients undergoing dysplasia-directed SC approach bear less clinically active disease as compared with those undergoing SC for active stenotic or fistulizing disease, and thus while not statistically significant, the lower risk of postoperative CD recurrence would seem logical.

Across the cohort, there was no statistical difference in sex, age at diagnosis, or smoking status. Prior studies demonstrate female sex, history of perianal disease, and age <18 years at time of resection to independently predict recurrence following SC for CD.^16^^,^^17^ Polle et al^17^ found female sex (OR, 12.5) and perianal disease (OR, 13.9) as independent risk predictors for disease recurrence. Martel et al^16^ and Pellino et al^18^ illustrated a risk for clinical and operative recurrence in patients <18 years of age at time of SC (hazard ratio, 2.8). It should be noted that both studies were conducted retrospectively within the early age of biologics. Furthermore, Scaringi et al^19^ showed a measurable risk for operative recurrence after SC across a longitudinal cohort (31% of patients, of which 66% required subsequent total colectomy). Pellino et al^18^ demonstrated perianal disease as an adverse predictor across a robust multicenter European cohort comparing outcomes of SC against total colectomy (hazard ratio, 1.9). Of note, our study did not reveal any impact of perianal disease history on recurrence. We query if this may relate to a more modern approach to the medical management of perianal disease with anti-TNFα inhibitors, possibly lessening referral for SC. We did, however, find a trend for increased rate of recurrence in those individuals undergoing SC prior to 40 years of age.

Current guidelines support the use of infliximab, adalimumab, ustekinumab, and vedolizumab in the induction of remission for CD.^20^^,^^21^ The role for TNFα inhibitors or other biologic use in the postoperative phase has been assessed, although this has typically looked at rates of postoperative recurrence after ileocolonic resection.^22–29^ A paucity of data persists relating to the use of anti-TNFα inhibitors amid a CD-directed SC surgical approach. Notably, Pellino et al^18^ did demonstrate an increased risk of operative recurrence in patients who were not placed on biologic therapy after total colectomy or SC. We assessed the use postoperative prophylactic TNFα inhibitors and other biologics. No signal was seen for other biologics, though the sample size was very small. The proactive use of TNFα inhibitors for prophylactic purposes showed a protective effect for the development of postoperative recurrence. Further, most patients who received postoperative prophylaxis with anti-TNFα agents were kept on the same agent prior to the surgical intervention in the postoperative phase. This aligns with recent demonstration in ileocolonic resection of a protective effect of anti-TNFα inhibitor prophylaxis along with most the meaningful use among anti-TNFα inhibitor–experienced patients.^15^

While multicenter recruitment does promote heterogeneity across the cohort, our study did have several limitations. Chiefly, the retrospective nature may permit confounding patient- or center-specific factors to go unrecognized. Furthermore, variability within operative approach and specifically the previously cited challenge of classifying mixed operative approach may allow unrecognized site-specific factors to confound the results. While able to identify those patients receiving postoperative biologic prophylaxis (both with and without subsequent disease recurrence), a small subset of patients newly received postoperative biologics for unclear indication. We suspect that this may relate to extraintestinal manifestations or residual or perianal disease, although we are unable to comment further. This certainly could allow for unrecognized nuances within postoperative clinical course and potentially underestimate the use of prophylactic biologics. Last, due to limitations in retrospective review of endoscopic documentation, we are unable to further characterize type and severity of postoperative recurrence. Future prospective work with standardized endoscopic scoring may allow for a more effective stratification of postcolectomy recurrence.

To our knowledge, this study represents one of the largest multicenter studies to examine postoperative endoscopic recurrence among Crohn’s patients undergoing SC in the biologic era. Compared with other types of CD-directed surgery, we demonstrate a markedly high rate of postoperative recurrence after SC, 63.9% at a median of 17 months. Definitive predictors of patients at highest risk for postoperative recurrence remain unclear, and a continued assessment is needed of SC varieties and perioperative disease–directed therapies on both short- and long-term outcomes. Our data support a meaningful protective effect and need for proactive anti-TNFα inhibitor prophylaxis amid SC. CD patients undergoing SC for indication of malignancy or those undergoing an APR procedure have low rates of recurrence and may not need biologic prophylaxis.
